# Secreted Protein Acidic and Rich in Cysteine as an Exercise-Induced Gene: Towards Novel Molecular Therapies for Immobilization-Related Muscle Atrophy in Elderly Patients

**DOI:** 10.3390/genes13061014

**Published:** 2022-06-04

**Authors:** Abdelaziz Ghanemi, Mayumi Yoshioka, Jonny St-Amand

**Affiliations:** 1 Department of Molecular Medicine, Faculty of Medicine, Laval University, Quebec, QC G1V 0A6, Canada; abdelaziz.ghanemi@crchudequebec.ulaval.ca; 2 Functional Genomics Laboratory, Endocrinology and Nephrology Axis, CHU de Québec-Université Laval Research Center, Quebec, QC G1V 4G2, Canada; mayumi.yoshioka@crchudequebec.ulaval.ca

**Keywords:** SPARC, muscle atrophy, immobilization, ageing

## Abstract

Long periods of immobilization, among other etiologies, would result is muscle atrophy. Exercise is the best approach to reverse this atrophy. However, the limited or the non-ability to perform the required physical activity for such patients and the limited pharmacological options make developing novel therapeutic approaches a necessity. Within this context, secreted protein acidic and rich in cysteine (*SPARC*) has been characterized as an exercise-induced gene. Whereas the knock-out of this gene leads to a phenotype that mimics number of the ageing-induced and sarcopenia-related changes including muscle atrophy, overexpressing SPARC in mice or adding it to muscular cell culture produces similar effects as exercise including enhanced muscle mass, strength and metabolism. Therefore, this piece of writing aims to provide evidence supporting the potential use of *SPARC*/SPARC as a molecular therapy for muscle atrophy in the context of immobilization especially for elderly patients.

The increased number of hospitalized individuals lead to the development of various fields aiming to improve and optimize the healthcare within hospitals [1,2,3,4,5]. Patients admitted to hospitals have, beside treating the reasons of their admission, also to face other challenges such as possible nosocomial infections [6], bedsores [7,8] and musculoskeletal atrophy. Furthermore, post-hospitalization recovery of the mobility remains a challenge due to the immobilization (bed rest)-induced muscle atrophy. Such bed resting (immobilization) does not only lead to muscle atrophy, but also reduces both muscle strength as well as key regulators of mitochondrial biogenesis/remodeling and activity; it also alters genes expression and leads to metabolic decline including insulin resistance [9,10,11,12]. Bed resting also impacts bones and reduces their mineral density [13]. Cardiovascular complications and cardiac atrophy have also been reported following bed rest [14,15]. The consequences on the locomotor system impact the mass, the strength and the metabolism. Thus, patients, especially elderly people, have a difficulty to return to normal life after a certain period of bed rest caused by hospitalization or immobilization mainly because of muscle atrophy. In addition, ageing reduces both myogenesis [16] and skeletal muscle stem cells regenerative capacity [17]. Ageing also has specific genes expression signature [18,19] and shares numerous patterns with obesity such as epigenetic changes, inflammation and metabolic impairments [20]. These elements show the seriousness of the clinical outcomes of combining immobilization and ageing. The increased hospitalization rate represents one of the features of the current ongoing COVID-19 pandemic especially among the elderly patients who are already vulnerable. Intensive care unit patients (also increased with COVID-19) have more muscle loss especially with long hospitalization periods [21]. Furthermore, the elderly population has a limited physical activity within their lifestyle. Indeed, many of them spend long periods of immobilization due to some diseases or accidents requiring bed rest or hospitalizations. Ageing is another factor which, either independently or combined to immobilization, significantly contributes to the muscle and bone loss. Sarcopenia is an age-related decline in muscles mass and strength [22]. Age-related comorbidities such as chronic heart failure [23] and chronic obstructive pulmonary diseases [24] accelerate sarcopenia [25]. Clinically, sarcopenia epidemiological profile is increasing and enhances mortality [26] especially with the increasing number of elderly people who develop a poor lifestyle (reduced activity, unhealthy diet, etc.).

Muscle atrophy includes protein degradation, mitochondrial dysregulation and inflammation among its key biological features [27,28,29]. Biological markers suggested for sarcopenia [25,30] would represent significant diagnosis tools for muscle atrophy as well. Both muscle atrophy and bone loss (key tissues of the locomotor system) can be reversed by physical activity [31,32]. Exercise is known for its benefits in respect to muscle function and metabolism including as sarcopenia treatment [33,34,35,36]. The effects of exercise, including pre-training, on muscle atrophy and recovery has also been highlighted [37,38,39]. Indeed, muscle atrophy could be prevented by exercise [40], including a pretraining as suggested by electrical stimulation studies [41,42]. Exercise represents the main treatment approach and electrical stimulation and “cytoprotective” dietary interventions are also used against muscle atrophy [43,44]. Other therapeutic options represent potential approaches such as gene therapy and epigenetic drugs [45,46,47]. Pharmacological therapies, however, remain limited to some growth factors among which we cite insulin, ghrelin/IGF-1 analogues, testosterone and growth hormone [45,47]. The limitation in therapeutic options is in part due to the limited knowledge on the underlying molecular pathways and physiopathological processes.

To reveal such mechanism and deepen our understating of these immobilization-induced atrophy, animal models of immobilization-induced muscle atrophy (rats, mice, rabbit) [26,48,49,50] have been developed. Mice remain the best choice due to their affordable cost, genetic manipulation possibilities and short lifespan; in addition to the ageing process similarities, they share with humans [51,52,53,54,55]. Cast immobilization is the most used because it mimics prolonged immobilization in terms of muscle atrophy [56,57]. The immobilization also induces bone loss in both growing and adult mice [58]. Thus, such immobilization alters the two main parts of the locomotor system, muscles and bones. Bone and muscle mass are reduced with immobilization in which various biological changes such as inflammation, increased muscle RING finger 1 and mRNA contents of polyubiquitin and the ubiquitin ligases muscle atrophy F-box along with reduced rapamycin complex 1 signaling and reducing the myofiber size were reported [49,57,59,60,61]. Immobilization-induced muscle loss depends on factors such as age and sex. For instance, unilateral hindlimb immobilization in rats of different ages leads to a muscle mass loss inversely proportional to age [61]. The difference between male and female in muscle atrophy depends on whether it is aging-induced or inflammation-based [21]. In addition, hindlimb unloading induced more muscle loss in female rats than in males [62]. This could indicate that females would be more impacted by bed resting. Such age and sex differences suggest the need to adapt the treatment (nature and intensity) based on these two factors as well.

Functional genomics and genes expression patterns can lead to the identification of potential novel therapies for the atrophy resulting from the immobilization including during bed rest. Herein, we focus on the gene secreted protein acidic and rich in cysteine (*SPARC/Sparc*). SPARC is a non-collagenous protein that is abundant in mineralized tissues [63]. It is expressed in various situations in which tissues renewal and cell remodeling occur (exercise, regeneration, obesity, cancer, inflammation, etc.) [64]. It is also associated with cell turnover, remodeling and tissue repair [65]. Based on this expression pattern, we and others previously suggested using SPARC as a molecular physiological and pathological biomarker [64,66]. SPARC, also known as osteonectin or basement membrane-40 (BM-40) [67], has a calcium and collagen binding property [68]. It is a secreted protein that comprises three distinct structural domains [69] and its biosynthesis is regulated by various growth factors and cytokines [70,71,72]. As exemplified below, SPARC plays important roles in muscles biology. This gene was initially characterized as induced by exercise [73,74], potentially mediating exercise-induced muscle phenotype changes [75] and as up-regulated during skeletal muscle regeneration [76]. *Sparc* overexpression mimics exercise, including enhancing muscle mass, strength, metabolism as well as ameliorating glycemia [77]. SPARC is expressed both in fetal and neonatal muscle and following muscle damage as well [78]. Adding SPARC to muscle C2C12 (myoblast cell) culture increased myoblasts differentiation in addition to myogenic and mitochondrial proteins expression [79]. Moreover, SPARC plays roles in muscle stiffness maintenance [80], muscle morphological change [81] and promotes muscle progenitor cells myogenic differentiation in vitro [80]. On the other hand, *Sparc* expression [82] and muscle mass [83] decline with ageing. Such age-related decline in SPARC expression would explain why SPARC downregulation using siRNA reduced myogenesis in young rats skeletal muscle progenitor cells (SMPCs) but had little effect in SMPCs from old rats [84] since old rats would already have low SPARC levels. A resistance to SPARC with age is suggested by the fact that exogenous SPARC improved differentiation in young SMPCs, but exogenous SPARC did not affect old SMPCs [84]. This indicate that SPARC would be combined to other therapies which require further investigation especially with the other effects SPARC has on muscles as we detail below.

Furthermore, *Sparc* KO leads to a phenotype that mimics number of the ageing-induced and sarcopenia-related changes including muscle atrophy with a decrease in muscle mass, strength and metabolism [77]. Small interfering RNA (siRNA)-mediated transient suppression of SPARC leads to muscle atrophy [59] and myofibers atrophic changes [80]. Anti-SPARC antibodies reduced C2C12 differentiation and decreased myogenin expression [79,81]. These suggest that the muscle atrophy could have the decline of SPARC expression as one of its key underlying pathways. Thus, SPARC decline would be implicated within both sarcopenia as well as ageing process that impacts muscles as well.

Such similarities between SPARC impacts on muscles (enhanced functional, structural and metabolic properties) and the exercise-induced muscle changes hypothesize that exercise effects are mediated, at least in part, by SPARC. Therefore, increasing *SPARC* expression (gene therapy) or administering SPARC protein would possibly lead to exercise-like effects similarly to those seen in mice overexpressing *Sparc* [77]. This would result in increasing muscles mass, strength and metabolism and counteract the atrophy resulting from hospitalization (immobilization), ageing, or more importantly hospitalization of elderly patients (combines ageing and immobilization). Indeed, hospitalized patients have long periods of immobilization during which they are not able to perform physical activity. Similarly, elderly individuals usually have a limited ability to perform high amounts of exercise. Therefore, administering SPARC or inducing its expression could be an option to overcome these struggles by generating some of the exercise-induced effects without in fact performing exercise. As muscle atrophy is among the most important health problems for these patients (immobilized and/or aged), SPARC comes as a potential therapy as its specific impacts on muscles are well documents. Importantly, the literature also shows the divers beneficial properties and implications of SPARC including metabolic properties [85,86], anticancer [87], anti-inflammatory [88], collagen regulation in the heart [89], tissue repair and regeneration [90,91]. These SPARC properties allowed us to classify it as a regeneration factor [90] that would create a biological environment with optimum conditions for regeneration, muscle differentiation and growth properties.

The importance of SPARC in bones increases the potential of SPARC in managing the bed rest-induced atrophy since immobilization also leads to bone loss. Indeed, SPARC is important for bone formation, remodeling and regeneration [90]. *Sparc* KO mice develop osteopenia [92], decreased bone formation [93]. SPARC deficiency also affects bone marrow stromal function [94]. In addition, SPARC also plays roles in bone remodeling [95] and osteoblast maturation [67]. It also regulates hydroxyapatite crystals formation and growth [96] and influence osteogenic differentiation [97]. Furthermore, the implication of SPARC in other locomotor system constituents (such as ligaments [98,99] and tendons [100,101]) would make that treating with SPARC would not only improve muscle phenotype but could also have positive effects on the whole locomotion system. Therefore, SPARC administration might contribute to the maintenance of the musculoskeletal system responsible for the individual mobility during hospitalization and recovery periods. It is worth highlighting that increased SPARC expression has been reported in negative biological status such as metabolic disorders [102], rheumatoid arthritis [70], cancer [103], coronary artery disease [104] and intracranial aneurysms [105]. We have hypothesized that such expression would not indicate the involvement of SPARC in the pathogenesis or prognosis but rather represents an attempt to counteract the effects generated by such pathologies or disorders via the beneficial SPARC-mediated effects. Examples of SPARC counteracting inflammation [88] and cancer [87,106] would be two illustrations of such “regulatory feedback”.

Such approach can also be extended to those chronically bedridden, with physical disability or even space missions (microgravity environment) [107] as summarized in Figure 1. Evidence suggests that *Sparc* decline contributes to the muscle atrophy, ageing and the resulting phenotypes, whereas its overexpression induced by exercise would be a mechanism via which exercise corrects and improves muscle atrophy and ageing. Therefore, we suggested measuring exercise-induced SPARC/*SPARC*/*Sparc* expression as a molecular tool to optimize exercise therapy towards a personalized medicine [108] and also using SPARC as a potential “exercise substitute” [109]. Such measure could be applied to immobilized patients during a potential pre-training session aiming to counteract muscle atrophy. We believe that further animal and clinical studies could lead to a new generation of molecular therapies for muscle atrophy based on *SPARC* and permit the overcoming of this challenging atrophy resulting from hospitalization, immobility and ageing. The best option, when available, is to rather focus on exercise-induced SPARC as a possible treatment and we emphasize that further studies are needed to further map the mechanistic links between exercise, the exercise-induced myokines (including SPARC) and the exercise induced effects.

## Figures and Tables

**Figure 1:**
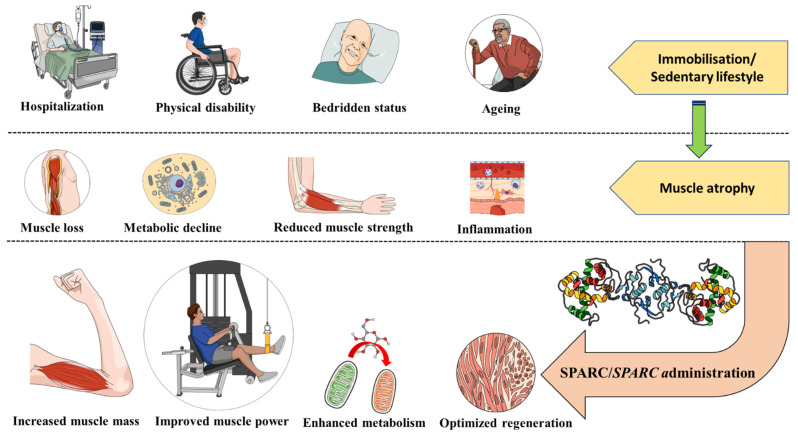
Secreted Protein Acidic and Rich in Cysteine (SPARC/*SPARC*) as a muscle atrophy therapy. Situations such as hospitalization, physical disability or being bedridden represent an immobilization that might lead to muscle atrophy. Ageing (usually accompanied with a sedentary lifestyle) is another risk factor for the muscle atrophy. SPARC properties of enhancing muscles mass, strength and metabolism are towards counteracting muscle atrophy and highlight SPARC/*SPARC* (protein administration or gene therapy) as a molecular therapy for muscle atrophy.

## Data Availability

Not applicable.

## References

[1] Wong H.J., Morra D. (2011). Excellent hospital care for all: Open and operating 24/7. J. Gen. Intern. Med..

[2] Lopez M.A., Hall M., Auger K.A., Bettenhausen J.L., Colvin J.D., Cutler G.J., Fieldston E., Macy M.L., Morse R., Raphael J.L. (2020). Care of Pediatric High-Cost Hospitalizations Across Hospital Types. Hosp. Pediatr..

[3] Copnell B., Hagger V., Wilson S.G., Evans S.M., Sprivulis P.C., Cameron P.A. (2009). Measuring the quality of hospital care: An inventory of indicators. Intern. Med. J..

[4] Buttigieg S.C., Abela L., Pace A. (2018). Variables affecting hospital length of stay: A scoping review. J. Health Organ. Manag..

[5] Hua M., Lu Y., Ma X., Morrison R.S., Li G., Wunsch H. (2020). Association Between the Implementation of Hospital-Based Palliative Care and Use of Intensive Care During Terminal Hospitalizations. JAMA Netw. Open.

[6] Liu J.Y., Dickter J.K. (2020). Nosocomial Infections: A History of Hospital-Acquired Infections. Gastrointest. Endosc. N. Am..

[7] Alwasel A., Alossimi B., Alsadun M., Alhussaini K. (2021). Bedsores Management: Efficiency Simulation of a New Mattress Design. Healthcare.

[8] Bluestein D., Javaheri A. (2008). Pressure ulcers: Prevention, evaluation, and management. Am. Fam. Physician.

[9] Buso A., Comelli M., Picco R., Isola M., Magnesa B., Pišot R., Rittweger J., Salvadego D., Šimunič B., Grassi B. (2019). Mitochondrial Adaptations in Elderly and Young Men Skeletal Muscle Following 2 Weeks of Bed Rest and Rehabilitation. Front. Physiol..

[10] LeBlanc A., Gogia P., Schneider V., Krebs J., Schonfeld E., Evans H. (1988). Calf muscle area and strength changes after five weeks of horizontal bed rest. Am. J. Sports Med..

[11] Mahmassani Z.S., Reidy P.T., McKenzie A.I., Stubben C., Howard M.T., Drummond M.J. (2019). Age-dependent skeletal muscle transcriptome response to bed rest-induced atrophy. J. Appl. Physiol..

[12] Dirks M.L., Smeets J.S.J., Holwerda A.M., Kouw I.W.K., Marzuca-Nassr G.N., Gijsen A.P., Holloway G.P., Verdijk L.B., van Loon L.J.C. (2019). Dietary feeding pattern does not modulate the loss of muscle mass or the decline in metabolic health during short-term bed rest. Am. J. Physiol. Endocrinol. Metab..

[13] Bloomfield S.A. (1997). Changes in musculoskeletal structure and function with prolonged bed rest. Med. Sci. Sports Exerc..

[14] Perhonen M.A., Franco F., Lane L.D., Buckey J.C., Blomqvist C.G., Zerwekh J.E., Peshock R.M., Weatherall P.T., Levine B.D. (2001). Cardiac atrophy after bed rest and spaceflight. J. Appl. Physiol..

[15] Dittmer D.K., Teasell R. (1993). Complications of immobilization and bed rest. Part 1: Musculoskeletal and cardiovascular complications. Can. Fam. Physician.

[16] Nakamura K., Yamanouchi K., Nishihara M. (2014). Secreted protein acidic and rich in cysteine internalization and its age-related alterations in skeletal muscle progenitor cells. Aging Cell.

[17] Sousa-Victor P., Muñoz-Cánoves P. (2016). Regenerative decline of stem cells in sarcopenia. Mol. Asp. Med..

[18] Melouane A., Ghanemi A., Aubé S., Yoshioka M., St-Amand J. (2018). Differential gene expression analysis in ageing muscle and drug discovery perspectives. Ageing Res. Rev..

[19] Melouane A., Ghanemi A., Yoshioka M., St-Amand J. (2019). Functional genomics applications and therapeutic implications in sarcopenia. Mutat. Res. Rev. Mutat. Res..

[20] Ghanemi A., Yoshioka M., St-Amand J. (2021). Ageing and Obesity Shared Patterns: From Molecular Pathogenesis to Epigenetics. Diseases.

[21] Rosa-Caldwell M.E., Greene N.P. (2019). Muscle metabolism and atrophy: Let’s talk about sex. Biol. Sex Differ..

[22] Nakamura K., Yamanouchi K., Nishihara M. (2014). Transdisciplinary Approach for Sarcopenia. Molecular mechanism of sarcopenia: The role of skeletal muscle niche component SPARC in the regulation of myogenesis and adipogenesis and its alteration with age. Clin. Calcium..

[23] Li H., Hastings M.H., Rhee J., Trager L.E., Roh J.D., Rosenzweig A. (2020). Targeting Age-Related Pathways in Heart Failure. Circ. Res..

[24] Easter M., Bollenbecker S., Barnes J.W., Krick S. (2020). Targeting Aging Pathways in Chronic Obstructive Pulmonary Disease. Int. J. Mol. Sci..

[25] Qaisar R., Karim A., Muhammad T., Shah I., Khan J. (2021). Prediction of sarcopenia using a battery of circulating biomarkers. Sci. Rep..

[26] Baek K.W., Jung Y.K., Kim J.S., Park J.S., Hah Y.S., Kim S.J., Yoo J.I. (2020). Rodent Model of Muscular Atrophy for Sarcopenia Study. J. Bone Metab..

[27] Bonaldo P., Sandri M. (2013). Cellular and molecular mechanisms of muscle atrophy. Dis. Mech..

[28] Ji L.L., Yeo D. (2019). Mitochondrial dysregulation and muscle disuse atrophy. F1000Res.

[29] Tuttle C.S.L., Thang L.A.N., Maier A.B. (2020). Markers of inflammation and their association with muscle strength and mass: A systematic review and meta-analysis. Ageing Res. Rev..

[30] Kwak J.Y., Hwang H., Kim S.K., Choi J.Y., Lee S.M., Bang H., Kwon E.S., Lee K.P., Chung S.G., Kwon K.S. (2018). Prediction of sarcopenia using a combination of multiple serum biomarkers. Sci. Rep..

[31] Miokovic T., Armbrecht G., Gast U., Rawer R., Roth H.J., Runge M., Felsenberg D., Belavý D.L. (2014). Muscle atrophy, pain, and damage in bed rest reduced by resistive (vibration) exercise. Med. Sci. Sports Exerc..

[32] Leblanc A.D., Schneider V.S., Evans H.J., Engelbretson D.A., Krebs J.M. (1990). Bone mineral loss and recovery after 17 weeks of bed rest. J. Bone Min. Res..

[33] Landi F., Marzetti E., Martone A.M., Bernabei R., Onder G. (2014). Exercise as a remedy for sarcopenia. Curr. Opin. Clin. Nutr. Metab. Care.

[34] Spaulding H.R., Selsby J.T. (2018). Is Exercise the Right Medicine for Dystrophic Muscle?. Med. Sci. Sports Exerc..

[35] Phu S., Boersma D., Duque G. (2015). Exercise and Sarcopenia. J. Clin. Densitom..

[36] Marzetti E., Calvani R., Tosato M., Cesari M., Di Bari M., Cherubini A., Broccatelli M., Savera G., D’Elia M., Pahor M. (2017). Physical activity and exercise as countermeasures to physical frailty and sarcopenia. Aging Clin. Exp. Res..

[37] He N., Ye H. (2020). Exercise and Muscle Atrophy. Adv. Exp. Med. Biol..

[38] Theilen N.T., Kunkel G.H., Tyagi S.C. (2017). The Role of Exercise and TFAM in Preventing Skeletal Muscle Atrophy. J. Cell Physiol..

[39] Salles J.I., Guimarães J.M., Filho G.M., Morrissey D. (2018). Effect of a specific exercise strategy on strength and proprioception in volleyball players with infraspinatus muscle atrophy. Scand. J. Med. Sci. Sports.

[40] Czerwinski S.M., Kurowski T.G., O’Neill T.M., Hickson R.C. (1987). Initiating regular exercise protects against muscle atrophy from glucocorticoids. J. Appl. Physiol..

[41] Baldi J.C., Jackson R.D., Moraille R., Mysiw W.J. (1998). Muscle atrophy is prevented in patients with acute spinal cord injury using functional electrical stimulation. Spinal Cord.

[42] Lake D.A. (1992). Neuromuscular electrical stimulation. An overview and its application in the treatment of sports injuries. Sports Med..

[43] Granic A., Sayer A.A., Robinson S.M. (2019). Dietary Patterns, Skeletal Muscle Health, and Sarcopenia in Older Adults. Nutrients.

[44] Anton S.D., Hida A., Mankowski R., Layne A., Solberg L.M., Mainous A.G., Buford T. (2018). Nutrition and Exercise in Sarcopenia. Curr. Protein. Pept. Sci..

[45] Urso M.L. (2009). Disuse atrophy of human skeletal muscle: Cell signaling and potential interventions. Med. Sci. Sports Exerc..

[46] Guasconi V., Puri P.L. (2008). Epigenetic drugs in the treatment of skeletal muscle atrophy. Curr. Opin. Clin. Nutr. Metab. Care.

[47] Ding S., Dai Q., Huang H., Xu Y., Zhong C. (2018). An Overview of Muscle Atrophy. Adv. Exp. Med. Biol..

[48] Son J.S., Kim J.H., Kim H.J., Yoon D.H., Kim J.S., Song H.S., Song W. (2016). Effect of resistance ladder training on sparc expression in skeletal muscle of hindlimb immobilized rats. Muscle Nerve.

[49] Caron A.Z., Drouin G., Desrosiers J., Trensz F., Grenier G. (2009). A novel hindlimb immobilization procedure for studying skeletal muscle atrophy and recovery in mouse. J. Appl. Physiol..

[50] Herbert R.D., Balnave R.J. (1993). The effect of position of immobilisation on resting length, resting stiffness, and weight of the soleus muscle of the rabbit. J. Orthop. Res..

[51] Yuan R., Peters L.L., Paigen B. (2011). Mice as a mammalian model for research on the genetics of aging. Ilar. J..

[52] Yuan R., Tsaih S.W., Petkova S.B., Marin de Evsikova C., Xing S., Marion M.A., Bogue M.A., Mills K.D., Peters L.L., Bult C.J. (2009). Aging in inbred strains of mice: Study design and interim report on median lifespans and circulating IGF1 levels. Aging Cell.

[53] Barreto G., Huang T.T., Giffard R.G. (2010). Age-related defects in sensorimotor activity, spatial learning, and memory in C57BL/6 mice. J. Neurosurg. Anesth..

[54] Graber T.G., Ferguson-Stegall L., Kim J.H., Thompson L.V. (2013). C57BL/6 neuromuscular healthspan scoring system. J. Gerontol. A Biol. Sci. Med. Sci..

[55] Parks R.J., Fares E., Macdonald J.K., Ernst M.C., Sinal C.J., Rockwood K., Howlett S.E. (2012). A procedure for creating a frailty index based on deficit accumulation in aging mice. J. Gerontol. A Biol. Sci. Med. Sci..

[56] Palus S., Springer J.I., Doehner W., von Haehling S., Anker M., Anker S.D., Springer J. (2017). Models of sarcopenia: Short review. Int. J. Cardiol..

[57] St-Amand J., Okamura K., Matsumoto K., Shimizu S., Sogawa Y. (2001). Characterization of control and immobilized skeletal muscle: An overview from genetic engineering. Faseb J..

[58] Friedman M.A., Zhang Y., Wayne J.S., Farber C.R., Donahue H.J. (2019). Single limb immobilization model for bone loss from unloading. J. Biomech..

[59] Nakamura K., Nakano S., Miyoshi T., Yamanouchi K., Nishihara M. (2013). Loss of SPARC in mouse skeletal muscle causes myofiber atrophy. Muscle Nerve.

[60] Krawiec B.J., Frost R.A., Vary T.C., Jefferson L.S., Lang C.H. (2005). Hindlimb casting decreases muscle mass in part by proteasome-dependent proteolysis but independent of protein synthesis. Am. J. Physiol. Endocrinol. Metab..

[61] Kelleher A.R., Pereira S.L., Jefferson L.S., Kimball S.R. (2015). REDD2 expression in rat skeletal muscle correlates with nutrient-induced activation of mTORC1: Responses to aging, immobilization, and remobilization. Am. J. Physiol. Endocrinol. Metab..

[62] Yoshihara T., Natsume T., Tsuzuki T., Chang S.W., Kakigi R., Sugiura T., Naito H. (2019). Sex differences in forkhead box O3a signaling response to hindlimb unloading in rat soleus muscle. J. Physiol. Sci..

[63] Rosset E.M., Bradshaw A.D. (2016). SPARC/osteonectin in mineralized tissue. Matrix Biol..

[64] Ghanemi A., Yoshioka M., St-Amand J. (2021). Secreted Protein Acidic and Rich in Cysteine as a Molecular Physiological and Pathological Biomarker. Biomolecules.

[65] Yan Q., Sage E.H. (1999). SPARC, a matricellular glycoprotein with important biological functions. J. Histochem. Cytochem..

[66] Kao S.C., Kirschner M.B., Cooper W.A., Tran T., Burgers S., Wright C., Korse T., van den Broek D., Edelman J., Vallely M. (2016). A proteomics-based approach identifies secreted protein acidic and rich in cysteine as a prognostic biomarker in malignant pleural mesothelioma. Br. J. Cancer.

[67] Delany A.M., Kalajzic I., Bradshaw A.D., Sage E.H., Canalis E. (2003). Osteonectin-null mutation compromises osteoblast formation, maturation, and survival. Endocrinology.

[68] Sage H., Johnson C., Bornstein P. (1984). Characterization of a novel serum albumin-binding glycoprotein secreted by endothelial cells in culture. J. Biol. Chem..

[69] Scavelli K., Chatterjee A., Rhee D.J. (2015). Secreted Protein Acidic and Rich in Cysteine in Ocular Tissue. J. Ocul. Pharm..

[70] Nakamura S., Kamihagi K., Satakeda H., Katayama M., Pan H., Okamoto H., Noshiro M., Takahashi K., Yoshihara Y., Shimmei M. (1996). Enhancement of SPARC (osteonectin) synthesis in arthritic cartilage. Increased levels in synovial fluids from patients with rheumatoid arthritis and regulation by growth factors and cytokines in chondrocyte cultures. Arthritis Rheum..

[71] Chandrasekhar S., Harvey A.K., Johnson M.G., Becker G.W. (1994). Osteonectin/SPARC is a product of articular chondrocytes/cartilage and is regulated by cytokines and growth factors. Biochim. Biophys. Acta.

[72] Fujita T., Shiba H., Van Dyke T.E., Kurihara H. (2004). Differential effects of growth factors and cytokines on the synthesis of SPARC DNA fibronectin and alkaline phosphatase activity in human periodontal ligament cells. Cell Biol. Int..

[73] Riedl I., Yoshioka M., Nishida Y., Tobina T., Paradis R., Shono N., Tanaka H., St-Amand J. (2010). Regulation of skeletal muscle transcriptome in elderly men after 6 weeks of endurance training at lactate threshold intensity. Exp. Gerontol..

[74] Ghanemi A., Melouane A., Yoshioka M., St-Amand J. (2020). Exercise and High-Fat Diet in Obesity: Functional Genomics Perspectives of Two Energy Homeostasis Pillars. Genes.

[75] Ghanemi A., Melouane A., Yoshioka M., St-Amand J. (2020). Exercise Training of Secreted Protein Acidic and Rich in Cysteine (Sparc) KO Mice Suggests That Exercise-Induced Muscle Phenotype Changes Are SPARC-Dependent. Appl. Sci..

[76] Petersson S.J., Jørgensen L.H., Andersen D.C., Nørgaard R.C., Jensen C.H., Schrøder H.D. (2013). SPARC is up-regulated during skeletal muscle regeneration and inhibits myoblast differentiation. Histol. Histopathol..

[77] Ghanemi A., Melouane A., Yoshioka M., St-Amand J. (2022). Secreted Protein Acidic and Rich in Cysteine (Sparc) KO Leads to an Accelerated Ageing Phenotype Which Is Improved by Exercise Whereas SPARC Overexpression Mimics Exercise Effects in Mice. Metabolites.

[78] Jørgensen L.H., Petersson S.J., Sellathurai J., Andersen D.C., Thayssen S., Sant D.J., Jensen C.H., Schrøder H.D. (2009). Secreted protein acidic and rich in cysteine (SPARC) in human skeletal muscle. J. Histochem. Cytochem..

[79] Melouane A., Carbonell A., Yoshioka M., Puymirat J., St-Amand J. (2018). Implication of SPARC in the modulation of the extracellular matrix and mitochondrial function in muscle cells. PLoS ONE.

[80] Omi S., Yamanouchi K., Nakamura K., Matsuwaki T., Nishihara M. (2019). Reduced fibrillar collagen accumulation in skeletal muscle of secreted protein acidic and rich in cysteine (SPARC)-null mice. J. Vet. Med. Sci..

[81] Cho W.J., Kim E.J., Lee S.J., Kim H.D., Shin H.J., Lim W.K. (2000). Involvement of SPARC in in vitro differentiation of skeletal myoblasts. Biochem. Biophys. Res. Commun..

[82] Aoi W., Naito Y., Takagi T., Tanimura Y., Takanami Y., Kawai Y., Sakuma K., Hang L.P., Mizushima K., Hirai Y. (2013). A novel myokine, secreted protein acidic and rich in cysteine (SPARC), suppresses colon tumorigenesis via regular exercise. Gut.

[83] Balachandran A., Krawczyk S.N., Potiaumpai M., Signorile J.F. (2014). High-speed circuit training vs hypertrophy training to improve physical function in sarcopenic obese adults: A randomized controlled trial. Exp. Gerontol..

[84] Nakamura K., Nakano S., Miyoshi T., Yamanouchi K., Matsuwaki T., Nishihara M. (2012). Age-related resistance of skeletal muscle-derived progenitor cells to SPARC may explain a shift from myogenesis to adipogenesis. Aging.

[85] Ghanemi A., Yoshioka M., St-Amand J. (2020). Secreted Protein Acidic and Rich in Cysteine: Metabolic and Homeostatic Properties beyond the Extracellular Matrix Structure. Appl. Sci..

[86] Ghanemi A., Melouane A., Yoshioka M., St-Amand J. (2019). Secreted protein acidic and rich in cysteine and bioenergetics: Extracellular matrix, adipocytes remodeling and skeletal muscle metabolism. Int. J. Biochem. Cell Biol..

[87] Ghanemi A., Yoshioka M., St-Amand J. (2020). Secreted protein acidic and rich in cysteine and cancer: A homeostatic hormone?. Cytokine.

[88] Ghanemi A., Yoshioka M., St-Amand J. (2020). Secreted protein acidic and rich in cysteine and inflammation: Another homeostatic property?. Cytokine.

[89] Harris B.S., Zhang Y., Card L., Rivera L.B., Brekken R.A., Bradshaw A.D. (2011). SPARC regulates collagen interaction with cardiac fibroblast cell surfaces. Am. J. Physiol. Heart Circ. Physiol..

[90] Ghanemi A., Yoshioka M., St-Amand J. (2021). Secreted Protein Acidic and Rich in Cysteine as A Regeneration Factor: Beyond the Tissue Repair. Life.

[91] Ghanemi A., Yoshioka M., St-Amand J. (2022). Exercise, Diet and Sleeping as Regenerative Medicine Adjuvants: Obesity and Ageing as Illustrations. Medicines.

[92] Mansergh F.C., Wells T., Elford C., Evans S.L., Perry M.J., Evans M.J., Evans B.A. (2007). Osteopenia in Sparc (osteonectin)-deficient mice: Characterization of phenotypic determinants of femoral strength and changes in gene expression. Physiol. Genom..

[93] Delany A.M., Amling M., Priemel M., Howe C., Baron R., Canalis E. (2000). Osteopenia and decreased bone formation in osteonectin-deficient mice. J. Clin. Investig..

[94] Luo Z., Zhou Y., Luo P., Zhao Q., Xiao N., Yu Y., Yan Q., Lu G., Cheng L. (2014). SPARC deficiency affects bone marrow stromal function, resulting in impaired B lymphopoiesis. J. Leukoc. Biol..

[95] Ribeiro N., Sousa S.R., Brekken R.A., Monteiro F.J. (2014). Role of SPARC in bone remodeling and cancer-related bone metastasis. J. Cell Biochem..

[96] Sodek J., Zhu B., Huynh M.H., Brown T.J., Ringuette M. (2002). Novel functions of the matricellular proteins osteopontin and osteonectin/SPARC. Connect. Tissue Res..

[97] Rowe D.W., Bilezikian J.P., Martin T.J., Clemens T.L., Rosen C.J. (2020). Chapter 61—Osteogenesis imperfecta. Principles of Bone Biology.

[98] Trombetta J.M., Bradshaw A.D. (2010). SPARC/osteonectin functions to maintain homeostasis of the collagenous extracellular matrix in the periodontal ligament. J. Histochem. Cytochem..

[99] Rosset E.M., Trombetta-eSilva J., Hepfer G., Yao H., Bradshaw A.D. (2017). SPARC and the N-propeptide of collagen I influence fibroblast proliferation and collagen assembly in the periodontal ligament. PLoS ONE.

[100] Gehwolf R., Wagner A., Lehner C., Bradshaw A.D., Scharler C., Niestrawska J.A., Holzapfel G.A., Bauer H.C., Tempfer H., Traweger A. (2016). Pleiotropic roles of the matricellular protein Sparc in tendon maturation and ageing. Sci. Rep..

[101] Wang T., Wagner A., Gehwolf R., Yan W., Passini F.S., Thien C., Weissenbacher N., Lin Z., Lehner C., Teng H. (2021). Load-induced regulation of tendon homeostasis by SPARC, a genetic predisposition factor for tendon and ligament injuries. Sci. Transl. Med..

[102] Kos K., Wilding J.P. (2010). SPARC: A key player in the pathologies associated with obesity and diabetes. Nat. Rev. Endocrinol..

[103] Chang C.-H., Yen M.-C., Liao S.-H., Hsu Y.-L., Lai C.-S., Chang K.-P., Hsu Y.-L. (2017). Secreted Protein Acidic and Rich in Cysteine (SPARC) Enhances Cell Proliferation, Migration, and Epithelial Mesenchymal Transition, and SPARC Expression is Associated with Tumor Grade in Head and Neck Cancer. Int. J. Mol. Sci..

[104] Takahashi M., Nagaretani H., Funahashi T., Nishizawa H., Maeda N., Kishida K., Kuriyama H., Shimomura I., Maeda K., Hotta K. (2001). The expression of SPARC in adipose tissue and its increased plasma concentration in patients with coronary artery disease. Obes. Res..

[105] Tan X., Li T., Zhu S., Zhong W., Li F., Wang Y. (2020). Induction of SPARC on Oxidative Stress, Inflammatory Phenotype Transformation, and Apoptosis of Human Brain Smooth Muscle Cells Via TGF-β1-NOX4 Pathway. J. Mol. Neurosci..

[106] Ma J., Gao S., Xie X., Sun E., Zhang M., Zhou Q., Lu C. (2017). SPARC inhibits breast cancer bone metastasis and may be a clinical therapeutic target. Oncol. Lett..

[107] Droppert P.M. (1993). A review of muscle atrophy in microgravity and during prolonged bed rest. J. Br. Interplanet. Soc..

[108] Ghanemi A., Yoshioka M., St-Amand J. (2021). Measuring Exercise-Induced Secreted Protein Acidic and Rich in Cysteine Expression as a Molecular Tool to Optimize Personalized Medicine. Genes.

[109] (2022). Ghanemi A, Yoshioka M, St-Amand J: Genetic Expression between Ageing and Exercise: Secreted Protein Acidic and Rich in Cysteine as a Potential “Exercise Substitute” Antiageing Therapy. Genes.

